# Autologous Blood Transfusion after Local Infiltration Analgesia with Ropivacaine in Total Knee and Hip Arthroplasty

**DOI:** 10.1155/2012/458795

**Published:** 2012-08-05

**Authors:** Torben Breindahl, Ole Simonsen, Peter Hindersson, Bjarne Brødsgaard Dencker, Mogens Brouw Jørgensen, Sten Rasmussen

**Affiliations:** ^1^Department of Clinical Biochemistry, Vendsyssel Hospital, Aalborg University, Bispensgade 37, 9800 Hjørring, Denmark; ^2^Orthopaedic Surgery Research Unit, Aalborg Hospital, Aarhus University, Sdr. Skovvej 15, 9000 Aalborg, Denmark; ^3^Department of Anaesthesiology, Vendsyssel Hospital, Aalborg University, Barfredsvej 83, 9900 Frederikshavn, Denmark

## Abstract

*Aims*. To study the safety of autotransfusion following local infiltration analgesia (LIA) with ropivacaine. *Background*. Knowledge of blood concentrations of ropivacaine after LIA and autotransfusion is crucial. However, very limited data are available for toxicological risk assessment. *Methods*. Autotransfusion was studied in patients after total knee arthroplasty (TKA: *n* = 25) and total hip arthroplasty (THA: *n* = 27) with LIA using 200 mg ropivacaine, supplemented with two postoperative bolus injections (150 mg ropivacaine). Drainage blood was reinfused within 6 h postoperatively. *Results*. Reinfusion caused a significant increase in the serum concentration of total ropivacaine for TKA from 0.54 ± 0.17 (mean ± SD) to 0.79 ± 0.20 **μ**g/mL (*P* < 0.001) and a nonsignificant increase for THA from 0.62 ± 0.17 to 0.63 ± 0.18 **μ**g/mL. The maximum free (unbound) concentration after reinfusion was 0.038 **μ**g/mL. Peak total and free venous ropivacaine concentrations after 8 h and 16 h postoperative bolus injections were 2.6 **μ**g/mL and 0.11 **μ**g/mL, respectively. All concentrations observed were below the threshold for toxicity and no side effects were observed. *Conclusion*. Autotransfusion of patients undergoing knee or hip arthroplasty after local infiltration analgesia with 200 mg ropivacaine can be performed safely, even supplemented with 8 h and 16 h postoperative bolus injections.

## 1. Introduction

Total knee and hip arthroplasty (TKA, THA) for osteoarthritis is still performed on broader indications even in elderly patients and in patients with previous or current medical conditions. In particular, comorbidity-like cardiovascular diseases and conditions with increased risk of bleeding or previous thromboembolic events are major challenges.

The use of intravenous tranexamic acid (TA) prior to the procedure in THA and before release of the tourniquet in TKA has reduced the per- and postoperative bleeding about 50% [1, 2]. In order to further reduce the need for allogeneic blood, reinfusion of drainage blood is recommended in procedures with significant per- or postoperative bleeding, including TKA and THA. Because blood loss is relatively limited when using TA, reinfusion is generally not required in TKA and THA, but highly relevant in situations with increased risk of bleeding, including conditions with contraindications to TA.

Intra- and periarticular local infiltration analgesia (LIA) have been introduced successfully in order to reduce postoperative pain and side effects to analgesics [3, 4]. Since drainage blood from arthroplasties treated with LIA might contain considerable amounts of the local analgesic drug, autologous blood transfusion in combination with LIA implies a risk of exceeding toxic serum concentration.

Only two minor studies have been published to show that reinfusion following TKA with LIA using ropivacaine can be performed safely [5, 6]. Hitherto, autotransfusion in combination with LIA supplemented by postoperative bolus injections as part of the pain management strategy has not been studied. In the present study, reinfusion was performed in a consecutive series of both hip and knee total arthroplasty, including ASA grade 1–3 patients, where ropivacaine was given as LIA in standard dose (3 mg/kg body weight, max. 200 mg) intraoperatively, supplemented by two intra-articular bolus injections of ropivacaine (150 mg).

## 2. Materials and Methods

### 2.1. Study Subjects

The study was approved on January 27, 2010 by the Human Research Ethics Committee in Region Northern Denmark (Ref. N-20090061). All patients enrolled in the study provided individual written, informed consent. During June 10, 2010 and November 29, 2010, 25 consecutive TKA and 27 consecutive THA patients were included in the study (Table 1). A sample of at least 24 patients was required to detect 1 SD difference in ropivacaine concentration [7] with a power of 90% at the 5% significance level.

### 2.2. Anaesthesia and Surgery

Single-shot spinal anaesthesia (0.5 mg/mL at a total of 13–18 mg bupivacaine) supplemented with 0.05–0.1 g fentanyl and 1–2 mg midazolam intravenously was used. Patients (*n* = 3), who refused to have spinal anaesthesia or had an anatomy not compatible with lumbar injection, received a general anaesthesia. All of the patients stayed overnight in the recovery department. Cefuroxime 1.5 g was given intravenously 0.5 h before surgery. Dalteparin (5000 IU per day) was started after surgery and administered the following 5 days. All patients received paracetamol 1 g four times daily, ketoprofen 200 mg daily, and immediate-release oxycodone 5 mg as required. Patients were mobilized to sitting or standing a few hours after surgery. Active flexion/extension exercise and walk training was initiated the next day.

In TKA, a tourniquet, a medial parapatellar approach, and a total cemented prosthesis with patellar resurfacing were used. THA was performed using a standard posterior approach and an uncemented prosthesis. Bone plugs were used to reduce bleeding and tranexamic acid (10 mg/kg) was given before release of the tourniquet in TKA, before the operation in THA, and again 3 hours later.

Both groups received LIA with 100 mL ropivacaine (2 mg/mL) or 3 mg/kg body weight, max. 200 mg. In TKA, the first 50 mL dose was given into the posterior part of the capsule, in the adjacent subcutaneous tissue and in the intercondylar area prior to cementing the prosthesis. The next 50 mL dose was given into the anterior part of the capsule, around the collateral ligaments and subcutaneously around the incision before closure of the capsule. A 12-gauge drain was positioned laterally in the joint cavity and a 20-gauge catheter was introduced separately from the drain into the posterior part of the joint cavity. In THA, 50 mL was infiltrated into the deep tissues (capsule, musculus gluteus medius, musculus gluteus maximus, and rotators). Before wound closure, the fascia, subcutaneous tissue, and the skin were infiltrated with the remaining 50 mL. The drain and the catheter were placed separately in the subfascial space. At 10 p.m. on the day of surgery (or 8 h postoperatively) and in the following morning at 7 a.m. (or 16 h postoperatively) intraarticular bolus injections of 150 mg ropivacaine (20 mL 7.5 mg/mL) were given. The catheter was removed immediately after the second bolus. The drain was closed 1 h following bolus injections and removed 2 h after the second bolus.

### 2.3. Blood Reinfusion

The Bellovac ABT autotransfusion system (Astra Tech, Mölndal, Sweden) was used. It consists of a blood collection suction bellow connected to an autologous transfusion bag with a 40 *μ*m filter connected to a transfusion set. Reinfusion was performed every time the drainage bag contained more than 500 mL and finally 6 h postoperatively. Hence, if the drainage volume was less than 500 mL, only one reinfusion procedure was performed.

### 2.4. Blood Sampling and Analysis

Reinfusion blood volumes (mL) were calculated by the formula: (mass of drainage bag before reinfusion (g)) minus (mass of drainage bag after reinfusion (g)) divided by the relative density of human blood (value used: 1.060 g/mL). A representative sample of the drainage blood was obtained from the transfusion bag after careful mixing immediately prior to reinfusion. Thus, the total amount of ropivacaine reinfused could be calculated by multiplying the estimated bag volume and the drain blood concentration. 

Venous blood samples were collected prior to reinfusion, immediately after reinfusion and in connection with the two bolus injections of 150 mg ropivacaine. Serum samples were analysed for total and free ropivacaine concentrations by High-Performance Liquid Chromatography (HPLC) with tandem mass spectrometry [7]. Determination of free ropivacaine was performed after ultrafiltration on fresh serum. The interassay coefficient of variation was 1.4 to 3.1% and accuracy (bias) was between −1.5 and 5.8%. Data below are presented as mean and range (min/max) or mean ± standard deviation (SD).

### 2.5. Statistical Methods

For statistical data analysis, SigmaPlot (version 12) was used (Systat software Inc., 2011). Data were tested for normality. If data were not normally distributed, a Mann-Whitney test was used.

## 3. Results and Discussion

Drainage blood volume after THA was significantly lower than after TKA (*P* = 0.007) (Table 1). Total serum concentration of ropivacaine before autotransfusion was 0.54 ± 0.17 *μ*g/mL after TKA and 0.62 ± 0.17 *μ*g/mL after THA. Autotransfusion caused a significant increase in total ropivacaine concentration to 0.79 ± 0.20 *μ*g/mL for TKA (*P* < 0.001) and a non-significant increase to 0.63 ± 0.18 *μ*g/mL for THA. Maximum amount of reinfused ropivacaine was 7.2 mg. The total serum concentration of ropivacaine measured at the end of autotransfusion did not exceed 1 *μ*g/mL (Table 1). The peak free concentration observed for all patients was 0.038 *μ*g/mL. No signs of toxicity were observed for any patient during the study.

Bolus injection I raised the total serum concentrations to maximum 1.9 *μ*g/mL with a peak-free concentration of 0.090 *μ*g/mL. Bolus injection II raised the total serum concentrations to maximum 2.6 *μ*g/mL with a peak free concentration of 0.11 *μ*g/mL.

**Figure 1:**
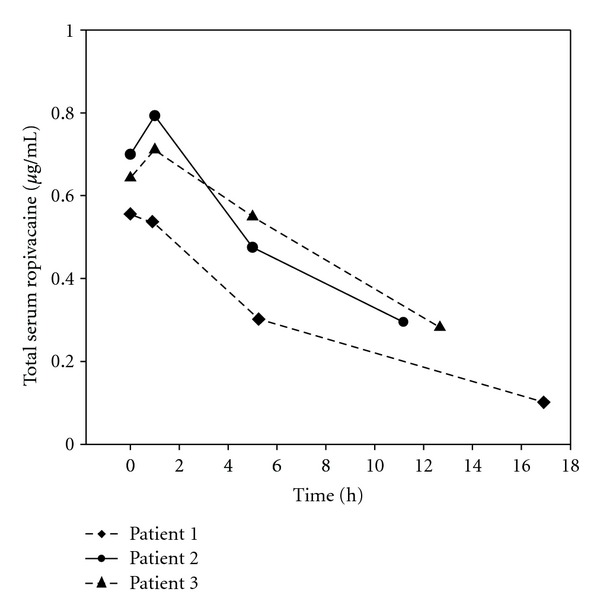
Serum concentration versus time profile of ropivacaine (*μ*g/mL) for 3 patients that did not receive postoperational bolus injections.

Three patients did not receive bolus I or bolus II injections due to blocked catheters. Their concentration versus time profile is shown in Figure 1.

### 3.1. Autotransfusion

Risk assessment of systemic toxicity after analgesia with ropivacaine is usually based on the work by Knudsen et al. on intravenous infusion of 10 mg/min in volunteers [8]. In this study, the total and free arterial plasma concentrations were consistently higher than corresponding venous concentrations during and up to 20 min after the end of infusion. Consequently, both arterial and venous blood thresholds for onset concentration of neurotoxicity were reported. The mean and ranges of total ropivacaine concentration were 4.3 *μ*g/mL (3.4–5.3) in arterial blood and 2.2 *μ*g/mL (0.5–3.2) in venous blood. In terms of free (unbound) concentrations the mean and ranges for neurotoxicity were 0.56 *μ*g/mL (0.34–0.85) and 0.15 *μ*g/mL (0.01–0.24) for arterial and venous blood, respectively. However, the toxicity thresholds suggested by Knudsen et al. with the broad concentration ranges are very difficult to apply in clinical practise. Numerous publications report high blood concentration levels of ropivacaine without toxic effect in patients. Venous serum concentration above 2.2 *μ*g/mL has been reported without any observable systemic toxicity after 120 h epidural infusion [9], intercostal blocks for thoracic surgery [10] fascia iliaca compartment block in children [11], and ultrasound-guided transversus abdominis plane block [12]. For slow systemic drug input, like epidural administration, it has been assumed that the peripheral venous free serum concentration and free arterial concentrations are similar [8, 13, 14]. This would also be the case for application of postoperative bolus injections. Consequently, the serum concentrations of the present study are related to the arterial toxic thresholds.

Convery et al. studied plasma concentrations after intraarticular injections of 100, 150, or 200 mg ropivacaine in TKA [15]. The peak ropivacaine concentration was 2.2 *μ*g/mL with an associated free ropivacaine concentration of 0.061 *μ*g/mL. The authors concluded that intraarticular injection is safe and that the 150 mg dose is most efficient for pain reduction. Later studies have verified the safety of LIA using 200 mg ropivacaine, with peak concentrations of 1.4 *μ*g/mL and peak free concentrations of 0.060 *μ*g/mL [16, 17]. The 200 mg dose was chosen in the present study in accordance with the studies previously mentioned and a regional, generally accepted maximum dose of 225 mg. The postoperative setup was performed according to the current national practise as recommended in reference programs by the Danish Orthopaedic Society. Later the benefit of LIA in THA and the additional pain reducing effect of LIA to a pain treatment protocol with gabapentin have been questioned [18–20].

To date, only four publications have addressed the safety of autotransfusion after periarticular infiltration techniques with ropivacaine (Table 2). Stringer et al. [21] studied drainage blood amounts of ropivacaine after TKA and THA and stated that autotransfusion should be safe. Gill et al. [6] reinfused drainage blood in 10 TKA patients after periarticular multimodal drug infiltration (PMDI) using ropivacaine (400 mg), ketorolac, epinephrine, and morphine, but only Parker et al. [5] have demonstrated the safety of reinfusion with 20 otherwise healthy subjects after TKA with LIA using ropivacaine only. Their mean amount injected was 1.9 mg (range: 0.4–2.6 mg) at an estimated mean infusion rate of 1.9 mg/h (range: 0.7–4.7 mg/h). Total venous serum concentrations were in the range from 0.5 to 1.5 *μ*g/mL with no adverse events. Their mean total serum concentration increased from 0.79 to 0.82 *μ*g/mL.

In 2010, we published preliminary data for 20 TKA and 10 THA patients using a new analytical method for quantification of total and free ropivacaine in serum and total ropivacaine in drainage blood [7]. These data show a mean total ropivacaine concentration of 0.64 *μ*g/mL (range: 0.30–1.1 *μ*g/mL) within 6 h, which is the time limit in which reinfusion should be initialised.

Data from the present study (Table 1) clearly show that autotransfusion after LIA with ropivacaine is safe, based on a comparison with the arterial threshold values by Knudsen et al. [8]. The maximum amount of ropivacaine reinfused (7.2 mg) is 8 times lower than the intravenous bolus threshold (60 mg) that produces mild symptoms of CNS toxicity [22]. Thus, the conclusion by Parker et al. [5] that autotransfusion implies a trivial risk is confirmed by this study.

### 3.2. Postoperative Bolus Injections

The timing and dose of ropivacaine in bolus injections in the present study are a routine adapted from a clinical trial by Toftdahl et al. [3]. However, the safety of the procedure regarding doses and intervals between bolus injections is still unclear [23].

Pharmacokinetic data after LIA is not available, but prolonged absorption from periarticular tissues has been observed, resulting in elevated serum concentrations for hours after surgery [15]. The ropivacaine concentrations measured in the present study stem from both LIA and autotransfusion, and the two postoperational bolus injections, which result in long drug detection times for the patients involved. We postulate that the high, total concentrations of ropivacaine, which peaked for one patient at 2.6 *μ*g/mL, were primarily caused by systemic drug release into the bloodstream after bolus injections. We also speculate that the large surface and vascularisation of the knee compartments can lead to an ultrafast absorption kinetic as recently reported for transversus abdominis plane blocks [24]. However, drug residues from LIA and autotransfusion are of minor importance, 6–18 h after surgery. This is also supported by the data for patients not receiving bolus injections (Figure 1). However, bolus injections given before 6 h after LIA and injection with higher amounts of ropivacaine and shorter intervals imply a potential risk. 

## 4. Conclusions

We conclude that autotransfusion of patients undergoing knee or hip arthroplasty after local infiltration analgesia with 200 mg ropivacaine can be performed safely, even supplemented with 8 h and 16 h postoperative bolus injections. All concentrations observed were below the estimated threshold for systemic toxicity and no side effects were observed after autotransfusion. However, infiltration techniques imply usage of large amounts of local anaesthetics and there is a lack of knowledge concerning safe reinjection protocols. Future studies concerning dose and timing for optimal bolus injections are relevant, as well as simulations, mathematical modelling, and population pharmacokinetic analyses.

## Figures and Tables

**Table 1:** Patient characteristics, autotransfusion, and drainage blood data.

Patient characteristics	TKA	THA
Number of patients enrolled (male, female)	25 (10,15)	27 (13,14)
Mean weight (kg) and range	85 (59–135)	80 (62–105)
Mean age and range (yr)	65 (45–88)	69 (47–86)
Number of patients given one autotransfusion	25	27
Number of patients given a second autotransfusion	1	1
Preinfusion data		
Mean serum concentration (*μ*g/mL) and range	0.54 (0.27–0.79)^a^	0.62 (0.32–0.97)
Max. free concentration (*μ*g/mL)	0.022	0.046
Postinfusion data (1-2 h)		
Mean serum concentration (*μ*g/mL) and range	0.79 (0.27–1.0)	0.63 (0.29–1.0)
Max. free concentration (*μ*g/mL)	0.038	0.035
Mean infusion rate (mg/h) and range	2.8 (0.42–7.2)	2.8 (0.48–12)
Drainage blood data		
Mean drainage blood volume (mL) and range	336 (93–977)	190 (66–408)
Mean drainage blood content of ropivacaine (mg) and range	2.6 (0.49–7.2)	1.9 (0.2–6.0)
Bolus injection I		
Mean time elapsed after surgery (h) and range	8 (5–10)	9 (4–13)
Mean serum concentration (*μ*g/mL) and range	1.2 (0.52–1.9)^b^	0.96 (0.48-1.7)^d^
Max. free concentration (*μ*g/mL)	0.090	0.060
Bolus injection II		
Mean time elapsed after bolus I (h) and range	10 (7–12)	8 (5–10)
Mean serum concentration (*μ*g/mL) and range	1.7 (0.53–2.6)^c^	1.5 (0.54–2.3)^e^
Max. free concentration (*μ*g/mL)	0.11	0.070

^a^ *n* = 23, 2 patients excluded due to late sampling after bolus injections.

^b^ *n* = 18, 7 TKA patients did not receive Bolus I.

^c^ *n* = 21, 4 TKA patients but not receive Bolus II.

^d^ *n* = 18, 9 THA patients did not receive Bolus I.

^e^ *n* = 19, 8 THA patients did not receive Bolus II.

**Table 2:** Comparison of studies in autotransfusion after LIA with ropivacaine.

Characteristic	Present study	Gill et al. [6]	Breindahl et al. [7]	Parker et al. [5]	Stringer et al. [21]
Number of patients	25 TKA, 27 THA	10 THA^∗^	20 TKA, 10 THA	20 TKA	15 TKA, 20 THA
Autotransfusion performed	Yes	Yes	No	Yes	No
Ropivacaine dose for LIA (mg)	200	400	200	150	360–400
Drainage blood volume (mL)	93–977	<1250	50–620	300–700	10–905
Drainage blood content of ropivacaine (mg)	<7.2	<6	0.28–12	<2.6	0.53–27
Infusion rate (mg/h)	0.42–12	—	—	0.7–4.7	—
Serum concentration before autotransfusion (*μ*g/mL)	<0.79	—	<1.1	<0.79	<2
Serum concentration after autotransfusion (*μ*g/mL)	<1.0	—	—	<1.5	—
Free ropivacaine concentration after autotransfusion (*μ*g/mL)	<0.038	—	—	—	—
Max. concentration after bolus injection I	1.9	—	1.5	—	—
Max. concentration after bolus injection II	2.6	—	—	—	—

^∗^ Peri-articular multimodal analgesia (PMDI).
